# Dissecting the Heterogeneity of Autism: Focus on Phelan-McDermid Syndrome

**DOI:** 10.1192/j.eurpsy.2022.1557

**Published:** 2022-09-01

**Authors:** C. Lamschtein, T.J. Chaffer

**Affiliations:** 1 Dalhousie, Psychiatry, Quispamsis, Canada; 2 McGill, Biology, Quispamsis, Canada

**Keywords:** Autism, Phelan-McDermid Syndrome, Phelan-McDermid Syndrome, autism

## Abstract

**Introduction:**

Autism spectrum disorders (ASD) are a group of neurodevelopmental disorders that show delays and deficits in the development of multiple brain functions, which are characterized by social communication, poor language development, and restricted and stereotyped patterns of interests and behaviours. ASD affects about 1-2 % of the population and are considered to be highly genetic in nature. Structural variations of chromosomes have been identified in some ASD individuals, most common on chromosome 7q, 15q and 22q.

**Objectives:**

1-To present a systematic literature review of the natural history of individuals with 22q13.3 deletion syndrome, Phelan-McDermid syndrome (PMS). PMS, increase awareness of different phenotypes 2- Correlation of clinical manifestations of PMS with hypothesized underlying biological mechanisms 3-Rational for novel treatments is inferred through translational neuroscience approaches.

**Methods:**

We have conducted a systematic literature review of the natural history of individuals with PMS, including both cross-sectional and long-term longitudinal analyses and correlation with hypothesized underlying biological mechanisms, including roles in regulation synaptic development, function, and plasticity. This systematic review includes the basis for a promising common pathway for ASD pathogenesis and the clinical implications of novel therapeutic strategies inferred through translational neuroscience approaches.

**Results:**

This systematic review, therefore, outlines the: (1) Pathophysiological basis and clinical manifestations of PMS; (2) PMS pre-clinical models and applications to ASD; and (3) clinical implications of novel therapeutic strategies.

**Conclusions:**

A promising common pathway for ASD pathogenesis and rational for novel treatments is inferred through translational neuroscience approaches. Neurobiological basis for lithium treatment is indeed supported by experimental results and current clinical findings.

**Disclosure:**

No significant relationships.

